# Establishment of a risk prediction model for prolonged mechanical ventilation after lung transplantation: a retrospective cohort study

**DOI:** 10.1186/s12890-023-02307-9

**Published:** 2023-01-10

**Authors:** Peigen Gao, Chongwu Li, Junqi Wu, Pei Zhang, Xiucheng Liu, Yuping Li, Junrong Ding, Yiliang Su, Yuming Zhu, Wenxin He, Ye Ning, Chang Chen

**Affiliations:** 1 Department of Thoracic Surgery, Shanghai Pulmonary Hospital, Tongji University School of Medicine, 507 Zhengmin Road, Shanghai, 200443 China; 2 Shanghai Engineering Research Center of Lung Transplantation, Shanghai, China

**Keywords:** Prolonged mechanical ventilation, Cold ischemia time, Primary graft dysfunction, Ventilation parameters, Prediction model

## Abstract

**Background:**

Prolonged mechanical ventilation (PMV), mostly defined as mechanical ventilation > 72 h after lung transplantation with or without tracheostomy, is associated with increased mortality. Nevertheless, the predictive factors of PMV after lung transplant remain unclear. The present study aimed to develop a novel scoring system to identify PMV after lung transplantation.

**Methods:**

A total of 141 patients who underwent lung transplantation were investigated in this study. The patients were divided into PMV and non-prolonged ventilation (NPMV) groups. Univariate and multivariate logistic regression analyses were performed to assess factors associated with PMV. A risk nomogram was then established based on the multivariate analysis, and model performance was further examined regarding its calibration, discrimination, and clinical usefulness.

**Results:**

Eight factors were finally identified to be significantly associated with PMV by the multivariate analysis and therefore were included as risk factors in the nomogram as follows: the body mass index (BMI, *P* = 0.036); primary diagnosis as idiopathic pulmonary fibrosis (IPF, *P* = 0.038); pulmonary hypertension (PAH, *P* = 0.034); primary graft dysfunction grading (PGD, *P* = 0.011) at T_0_; cold ischemia time (CIT *P* = 0.012); and three ventilation parameters (peak inspiratory pressure [PIP, *P* < 0.001], dynamic compliance [Cdyn, *P* = 0.001], and P/F ratio [*P* = 0.015]) at T_0_. The nomogram exhibited superior discrimination ability with an area under the curve of 0.895. Furthermore, both calibration curve and decision-curve analysis indicated satisfactory performance.

**Conclusion:**

A novel nomogram to predict individual risk of receiving PMV for patients after lung transplantation was established, which may guide preventative measures for tackling this adverse event.

**Graphic Abstract:**

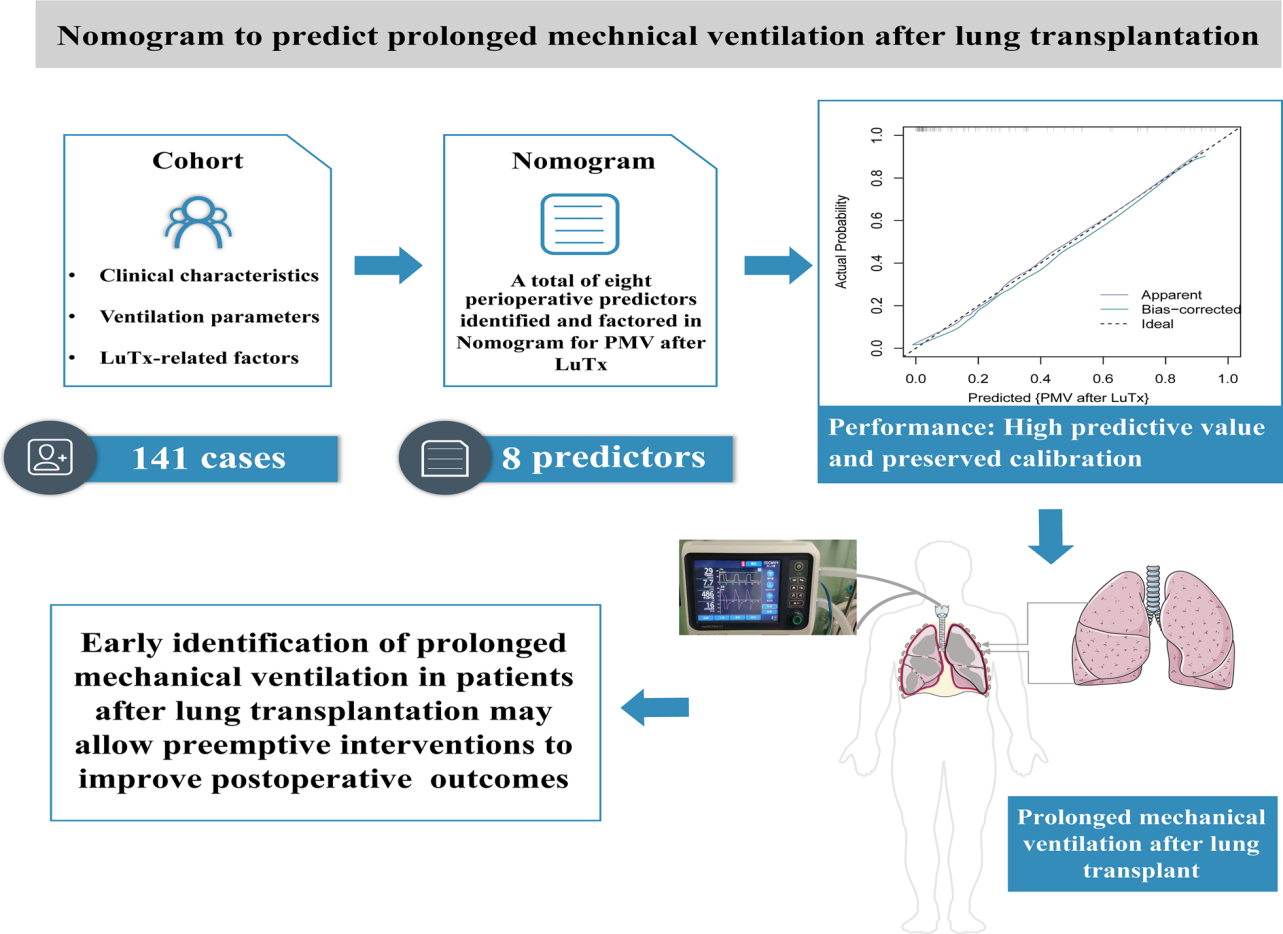

**Supplementary Information:**

The online version contains supplementary material available at 10.1186/s12890-023-02307-9.

## Background

More than 4,000 lung transplantations are currently performed worldwide per year [1]. However, the mortality and morbidity of lung transplant remain at relatively high levels compared with other solid organ transplantations [2]. Prolonged mechanical ventilation (PMV) is a prognostic marker for short-term adverse outcomes in patients after lung transplantation [3, 4]. Previous reports also show that PMV is associated with impaired long-term survival [5]. Thus, the discovery of predictors for PMV may assist in developing precautionary measures to ameliorate the high morbidity and mortality.

Primary graft dysfunction (PGD) is a form of acute lung injury that occurs in about 30% of patients after lung transplantation within 72 h, which can be characterized by hypoxemia and alveolar infiltrates in the allograft(s) [6]. PGD is reported as the most frequent cause of early death after lung transplantation and is also strongly correlated with other late outcomes [7–9]. Although the presence of PGD is associated with an increased duration of mechanical ventilation [9], recently, Schwarz and colleagues found that the value of PGD in predicting PMV was limited [3]. Instead, a model combing three ventilation parameters better predicted PMV. However, the predictive value of this model was still moderate, with an area under the curve (AUC) of 0.727 [3]. Thus, a more precise model is urgently required.

Apart from PGD and ventilation parameters, other factors, such as cold ischemia time (CIT), may also provide valuable information for predicting PMV, and CIT is closely associated with ischemia–reperfusion injury (IRI) [9, 10]. A recent study revealed that CIT is a risk factor for developing airway complications after lung transplantation [11], even though the correlation between CIT and PMV remains unknown. According to the latest guidelines, idiopathic pulmonary fibrosis (IPF) and idiopathic pulmonary arterial hypertension (IPAH) are strongly associated with risk for PGD in primary diagnoses[9]. However, to date, no research has considered the role of different primary diagnoses in predicting the early adverse events after lung transplant besides PGD. Therefore, our research aimed to develop a nomogram combing clinical variables (including primary diagnosis), CIT, PGD grading, and ventilation parameters to improve the predictive accuracy of PMV.

## Methods

### Study design and participants

With the Research Ethics Commission of Shanghai Pulmonary Hospital (Shanghai, China) approval (No. L20-352), we conducted a single-center, retrospective observational cohort study. Data from 146 patients who underwent lung transplantation at Shanghai Pulmonary Hospital were retrospectively extracted from electronic medical records between January 1, 2018, and February 1, 2022. The exclusion criteria included: patients with missing data; re-transplantation; postoperatively extended extracorporeal membrane oxygenation (ECMO) with clear chest radiographs (PGD ungradable) [6]. The use of postoperative extended ECMO was defined as the use of ECMO or re-use of ECMO to maintain life after arriving in the intensive care unit (ICU) after surgery [12]. According to the exclusion criteria, 141 patients who underwent lung transplant were included in our study cohort (Additional file 1: Figure S1).

### Data acquisition

The baseline characteristics and demographics of the patients (age, gender, BMI, smoking history, and documented pulmonary hypertension), primary diagnosis before the operation, the CIT, and length of mechanical ventilation were retrospectively collected from the medical case database of Shanghai Pulmonary Hospital. In this study, the length of mechanical ventilation was classified as PMV or non-prolonged mechanical ventilation (NPMV), with the threshold to be 72 h. The extubation criteria were as follows in our clinical practice as modified from the consensus on weaning from mechanical ventilation [13]: (1) successful spontaneous breathing trial lasting for 120 min; (2) hemodynamic stability; (3) No sedation or adequate mentation on sedation; (4) P/F ratio > 150 mm Hg with FiO_2_ ≤ 0.4, positive end-expiratory pressure ≤ 8 cm H_2_O. Patients who were extubated but needed reintubation within 72 h after lung transplantation were also included in the PMV group.

The ventilation parameters of T_0_, T_24_, T_48_, and T_72_ were also obtained. T_0_, T_24_, T_48_, and T_72_ were defined as the 2nd, 24th, 48th, and 72nd hours after the arrival at the ICU after transplantation, respectively. The ventilation parameters mainly included inhaled oxygen concentration fraction, arterial oxygen partial pressure, tidal volume (TV), peak inspiratory pressure (PIP), and positive end-expiratory pressure (PEEP). Dynamic compliance was calculated as tidal volume/(peak inspiratory pressure-positive end-expiratory pressure), while partial pressure of the oxygen fraction of inspired oxygen (P/F) ratio was calculated as arterial oxygen partial pressure (PaO_2_)/inhaled oxygen concentration fraction (FiO_2_).

The PGD diagnosis method in this study refers to the standard judgment of the International Society for Heart and Lung Transplantation (ISHLT) on PGD in 2016 [6]. Notably, patients receiving mechanical ventilation with FiO_2_ > 0.5 on nitric oxide > 48 h from lung transplant or using extracorporeal lung support (ECLS) with bilateral pulmonary edema on chest X-ray, which indicated ECLS is primarily hypoxemia, were classified as grade 3. In addition, using atomized prostacyclin or other drugs that may improve oxygenation did not affect PGD classification [14].

### Statistical analysis

The categorical variables were summarized as the absolute frequency and percentage, while the continuous variables were presented in the median and interquartile range (IQR). Fisher’s exact test and a non-parametric Mann–Whitney U test were performed to compare the categorical and continuous data, respectively. Subsequently, univariate and multivariate binary logistic regressions were calculated to test the effect of the PGD grading, CIT, and the ventilation parameters for predicting PMV [15]. Candidate factors with a univariate significance of *P* < 0.1 were selected for the multivariate analysis. The final multivariate model was displayed in the nomogram format to illustrate all the selected predictors of the individual risk of PMV. The linear relationship between the nomogram score and the length of mechanical ventilation was estimated by calculating Pearson’s correlation coefficient.

### Performance assessments

Bootstrapped calibration curves were used to assess the predictive probability of this model. The assessment determines whether the model is biased as a result of the overfitting of the model. The receiver operating characteristic curve (ROC) analysis was then performed to quantify the discrimination ability of the nomogram and the subjects included in it. The Bootstrap test was used to compare the area under the curve (AUC) of the different smoothed ROCs. The clinical utility was determined using decision-curve analysis (DCA), assessing the clinical net benefit associated with the use of the model [16]. The vertical axis, namely the net benefit (NB), was defined as the true positive rate minus the false positive rate over a range of threshold probability defining high risk. Each decision curve graphically illustrated the NB of the model and every indicator through a range of threshold probabilities of the outcome [17–19]. This study used R software (R-4.1.0) and SPSS v26.0 for the data analysis. The graphics were made with R or GraphPad Prism 9.0.0. Two-sided *P* values < 0.05 were used to declare statistical significance.

### Organ procurement statement

Voluntary organ donation by citizens has become the only legal source of deceased donor organ transplantation in China since starting on January 1, 2015, and the origins of all organs were registered in the Chinese organ donation system and have been traceable since that date. All the donation procedures were approved by The Institutional Ethics Committees of the Organ Procurement Organization (OPO). Donated lungs were prioritized to the listed candidates following the national organ allocation principles while considering the priority based on lung allocation score (LAS), a comprehensive measure of transplantation urgency and utility. Organ procurement was performed according to the standard protocol through the China Organ Transplant Response System (COTRS) [20]. Hence, it can be guaranteed that no organ used for lung transplantation during the study period was procured from executed prisoners.

## Results

### Patient characteristics

The demographical characteristics of our study cohort are presented in Table 1. Of the 141 patients in the cohort, the median age [interquartile range (IQR)] was 62 (56–66) years, with 103 (73.0%) male patients. Sixty-four (45.4%) patients received bilateral lung transplant, and seventy-seven (54.6%) patients received unilateral lung transplant. The most frequent diagnosis was idiopathic pulmonary fibrosis (IPF), followed by chronic obstructive pulmonary diseases (COPD) and interstitial lung disease (ILD). The median length of mechanical ventilation was 49 h, and 45 (31.9%) patients underwent PMV in the ICU after lung transplant. Other baseline characteristics of this retrospective cohort are listed in Table 1.

**Table 1:** Clinical characteristics of patients according to length of mechanical ventilation

Variables	Total (n = 141)	NPMV (n = 96)	PMV (n = 45)	*p* value
Age (years)	62 (56–66)	60 (56–66)	65 (57–69)	0.041
Gender				0.066
Male	103 (73.0)	75 (55.1)	28 (62.2)	
Female	38 (27.0)	21 (21.9)	17 (37.8)	
Smoking history				0.231
Never	40 (28.4)	24 (25.0)	16 (35.6)	
Ever	101 (71.6)	72 (75.0)	29 (64.4)	
BMI	21.5 (20.1–23.0)	20.5 (17.9–22.2)	22.7 (20.6–23.6)	**0.014**
Pulmonary hypertension	57 (40.4)	32 (33.3)	25 (55.5)	**0.010**
Pretransplant diagnosis				
LAM	4 (2.9)	4 (4.1)	0 (0)	0.320
COPD	37 (26.2)	28 (29.2)	9 (20.0)	0.385
IPF	56 (39.7)	29 (30.2)	27 (60.0)	**0.003**
ILD	23 (16.9)	18 (18.8)	5 (12.5)	0.076
Bronchiectasis	7 (5.1)	6 (6.3)	1 (2.5)	0.365
Pneumoconiosis	11 (10.6)	8 (8.3)	3 (7.5)	0.723
Others	3 (2.1)	3 (3.9)	0 (0)	0.320
Cardiac Comorbidities				
Arterial hypertension	25 (17.7)	15 (15.6)	10 (22.2)	0.170
Mild to moderate coronary artery disease	27 (19.1)	20 (20.8)	7 (15.5)	0.215
Heart failure	16 (11.3)	9 (9.3)	7 (15.6)	0.118
Atrial fibrillation	9 (6.4)	7 (7.3)	2 (4.4)	0.247
Type of transplant				0.858
Unilateral	77 (54.6)	53 (55.2)	24 (53.3)	
Bilateral	64 (45.4)	43 (44.8)	21 (46.7)	
Length of MV (hours)	49 (36–81)	38 (12–54)	94 (78–120)	** < 0.001**
CIT (hours)	7.0 (5.7–8.6)	4.0 (3.7–6.5)	8.5 (7.6–10.0)	** < 0.001**

Bold values indicate statistically significant (*P* < 0.05)

Continuous data are summarized as median and interquartile range (IQR). Categorical data are summarized as numbers and percentages

*BMI* body mass index; *LAM* lymphangioleiomyomatosis; *COPD* chronic obstructive pulmonary dysfunction; *ILD* interstitial lung disease; *IPF* idiopathic pulmonary fibrosis; *MV* mechanical ventilation; *CIT* cold ischemia time

### Comparison between the PMV and NPMV patients

Patients in the PMV group tended to be older (65 vs. 60 years, *p* = 0.041) and were more likely to have a higher BMI (22.7 vs. 20.5, *P* = 0.011) and longer CIT (*P* < 0.001) compared with the NPMV group. In addition, patients with primary diagnoses as IPF were more likely to undergo PMV than those diagnosed with other diseases (60.0% vs. 30.2%, *P* = 0.003). A similar trend was found for the presence of pulmonary hypertension (55.5% vs. 33.3%, *P* = 0.010, Table1), which was considered a complication of primary diagnoses. However, no statistically significant difference was found between the two groups regarding gender, smoking history, other diagnoses, and type of transplant.

As for the mechanical ventilation parameters at T_0_, more patients in the PMV group had controlled ventilation status than those in the NPMV group (95.0% vs. 79.1%, *P* = 0.015). Nevertheless, there was no significance in the detailed ventilation modes between the PMV and NPMV groups. Furthermore, patients who underwent PMV had a significantly higher peak inspiratory pressure (PIP, 19 vs. 16 cmH_2_O, *P* = 0.039) and lower dynamic compliance (Cdyn, 27.80 vs. 32.92, *P* = 0.018) and PaO_2_/FiO_2_ ratio (P/F ratio, 222 vs. 306, *P* = 0.041, Table 2). More detailed ventilation parameters are presented in the supplementary materials (Additional file 4: Table S1). We also investigated the difference in PGD grading between the subgroups. PGD grading was significantly higher in the PMV group, whereas the difference decreased over time (all *P* < 0.05, table 3). Additionally, no statistically significant differences were found in donor characteristics between the PMV and NPMV groups (Additional file 6: Table S3).

**Table 2:** Detailed Ventilation Parameters at T0 according to mechanical ventilation

Parameters	NPMV (n = 96)	PMV (n = 45)	*p* value
Ventilation status			**0.015**
Control ventilation	76 (79.1)	43 (95.6)	
Assisted ventilation	20 (20.8)	2 (4.4)	
Ventilation mode			0.763
Pressure controlled/assisted mode	18 (18.8)	3 (6.7)	
Pressure controlled ventilation mode	8 (8.3)	2 (4.4)	
Pressure assisted ventilation mode	10 (10.4)	1 (2.2)	
Volume controlled/assisted mode	78 (81.3)	42 (93.3)	
Volume controlled ventilation mode	68 (70.8)	41 (91.1)	
Volume assisted ventilation mode	10 (10.4)	1 (2.2)	
Ventilation parameters			
FiO_2_	0.60 (0.53–0.80)	0.40 (0.40–0.45)	0.095
PEEP (cm H_2_O)	5 (3–7)	5 (3–8)	0.125
Peak inspiratory pressure (cm H_2_O)	16 (14–20)	19 (14–22)	**0.039**
Tidal volume (ml)	394 (360–420)	360 (320–445)	0.116
Dynamic compliance (ml/cm H_2_O)	32.92 (15.66–41.31)	27.80 (21.12–43.56)	**0.018**
PaO_2_/FiO_2_ ratio	306 (282–390)	222 (150–332)	**0.041**

Bold values indicate statistically significant (*P* < 0.05)

Continuous data are summarized as median and interquartile range (IQR). Categorical and other data are summarized as numbers and percentages

**Table 3:** PGD grading of patients at T0, T24, T48, T72 after transplantation

PGD grades	NPMV n = 96	PMV n = 45	*p* value
T_0_ hours			** < 0.001**
PGD 0	58 (60.4)	11 (24.4)	
PGD 1	7 (7.3)	2 (4.4)	
PGD 2	24 (25.0)	3 (6.7)	
PGD 3	7 (7.3)	29 (64.4)	
T_24_ hours			**0.012**
PGD 0	60 (64.9)	14 (31.1)	
PGD 1	12 (12.5)	5 (11.1)	
PGD 2	18 (18.8)	5 (11.1)	
PGD 3	6 (6.3)	21 (46.6)	
T_48_ hours			**0.020**
PGD 0	66 (68.8)	20 (44.4)	
PGD 1	14 (14.6)	5 (11.1)	
PGD 2	12 (12.5)	5 (11.1)	
PGD 3	4 (4.2)	15 (33.3)	
T_72_ hours			**0.032**
PGD 0	81 (84.4)	28 (62.2)	
PGD 1	12 (12.5)	3 (6.7)	
PGD 2	3 (3.1)	2 (4.4)	
PGD 3	0 (0)	12 (17.5)	

Bold values indicate statistically significant (*P* < 0.05)

Categorical data are summarized as numbers and percentages

Prophylactic noninvasive ventilation after extubation was applied in 32 (22.7%) transplant recipients and the percentage of patients receiving noninvasive ventilation were similar between the NPMV and PMV groups (30.2% vs 44.4%; *P* = 0.272). Twenty-five (17.7%) patients underwent reintubation and the majority of patients underwent reintubation were in the PMV group (37.8% vs. 8.3%, *P* < 0.01, Additional file 7: Table S4).

### Logistic regression analyses

Possible correlations between PMV and thirteen parameters for the patients in this cohort were evaluated by univariate logistic regression. BMI, CIT, PGD grading at all times, pulmonary hypertension as a complication, primary diagnosis as IPF, and four ventilation parameters at T_0_ (ventilation status, PIP, P/F ratio and Cdyn) were identified as potential predictors for PMV (all *P* < 0.05), while age, gender and smoking history were considered not predictive. Further multivariate logistic regression identified 8 independent variables. BMI (odds ratio [OR] with 95% confidence interval [CI] 1.425[1.323–1.767]; *P* = 0.032), CIT (OR with 95% CI 1.777[1.065–2.889]; *P* = 0.012), PGD grading at T_0_ (OR with 95% CI 1.557[1.331–1.899]; *P* = 0.011), pulmonary hypertension (OR with 95% CI 1.894[1.243–3.001]; *P* = 0.034), primary diagnosis as IPF (OR with 95% CI 1.788[1.245–3.634]; *P* = 0.038), PIP (OR with 95% CI 1.961[1.211–2.747]; *P* < 0.001), P/F ratio (OR with 95% CI 0.991[0.980–0.996]; *P* = 0.015) and Cydn (OR with 95% CI 1.266[1.121–1.473]; *P* = 0.001) remained independent predictors of PMV (Table 4).

**Table 4:** Univariate and multivariate logistic regression analyses testing effects of perioperatively assessable variables on predicting PMV in 141 patients after LuTx

Characteristic	Univariable			Multivariable		
	OR	95% CI	*p* value	OR	95% CI	*p* value
Age, y	0.965	0.927–0.991	0.213	NA	NA	NA
BMI	1.213	1.219–1.444	**0.030**	1.425	1.323–1.767	**0.032**
Gender	0.957	0.927–0.994	0.445	NA	NA	NA
Smoking history Nonsmoker vs Smoker	1.204	1.162–1.231	0.145	NA	NA	NA
Pulmonary hypertension Normal vs High	2.706	1.278–5.845	**0.011**	1.894	1.243–3.001	**0.034**
Primary diagnose as IPF	3.001	1.643–6.153	**0.002**	1.788	1.245–2.634	**0.038**
PGDatT_0_	2.231	1.601–3.110	** < 0.001**	1.557	1.331–1.899	**0.011**
PGDatT_24_	1.599	1.082–2.361	**0.017**	0.205	0.044–0.958	0.054
PGDatT_48_	1.628	1.080–2.454	**0.024**	0.626	0.099–3.956	0.619
PGDatT_72_	1.510	1.518–2.262	**0.041**	2.007	0.427–9.430	0.378
CIT, h	2.068	1.537–2.783	** < 0.001**	1.777	1.065–2.889	**0.012**
Ventilation status CV vs AV	2.323	1.621–3.011	**0.003**	2.007	1.117–3.444	0.138
PIP	1.362	1.203–1.542	** < 0.001**	1.961	1.211–2.747	** < 0.001**
Cdyn	1.645	1.200–1.962	**0.001**	1.266	1.121–1.473	**0.001**
P/F ratio	0.993	0.989–0.998	**0.002**	0.991	0.981–0.996	**0.015**

Bold values indicate statistically significant (*P* < 0.05)

*BMI* body mass index; *IPF* idiopathic pulmonary fibrosis; *CIT* cold ischemia time; *CV*controlled ventilation; *AV*assisted ventilation; *TV* tidal volume; *PIP* peak airway pressure; *PEEP*positive end expiratory pressure; Cdyn, pulmonary dynamic compliance. Confidence interval; P/F ratio, PaO_2_/FiO_2_ ratio

In contrast, ventilation status and PGD grading at other times were not appropriate for inclusion in the final nomogram (all *P* > 0.05). We further investigated the prediction value of the donor factors using the univariate logistic regression analysis and we found no statistically significant differences in our results (Additional file 8: Table S5).

### Predictive nomogram for PMV

**Fig. 1:**
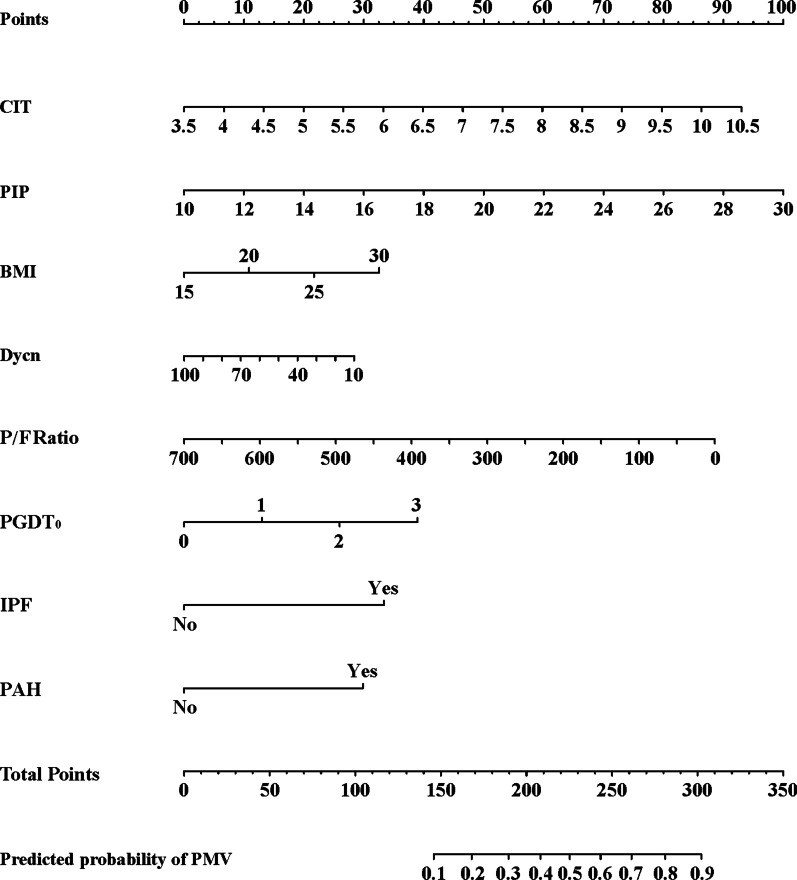
Risk prediction nomogram of logistic regression. Nomogram constructed to predict prolonged mechanical ventilation in lung transplant recipients after surgery. The included variables were cold ischemia time, ventilation parameters at T_0_ (including peak inspiratory pressure, tidal volume, dynamic compliance and oxygenation index), and PGD grade at T_0_. The full point density and risk density plots show their distribution. For category variables, their distribution is reflected by the size of the box. Rank the importance of each variable according to the standard deviation on the Nomogram scale. When using the Nomogram image, specific points (black spots) for each patient are located on each variable axis. Draw lines to determine the points received by each variable; The sum of these points is placed on the total point line and a line drawn down the risk line to obtain the total predicted risk of prolonged ventilation after surgery. CIT, cold ischemia time; PGDT_0_, primary graft dysfunction at T_0_; BMI, body mass index; Cydn, dynamic compliance; PIP, peak inspiratory pressure; PAH pulmonary hypertension; IPF, idiopathic pulmonary fibrosis

**Fig. 2:**
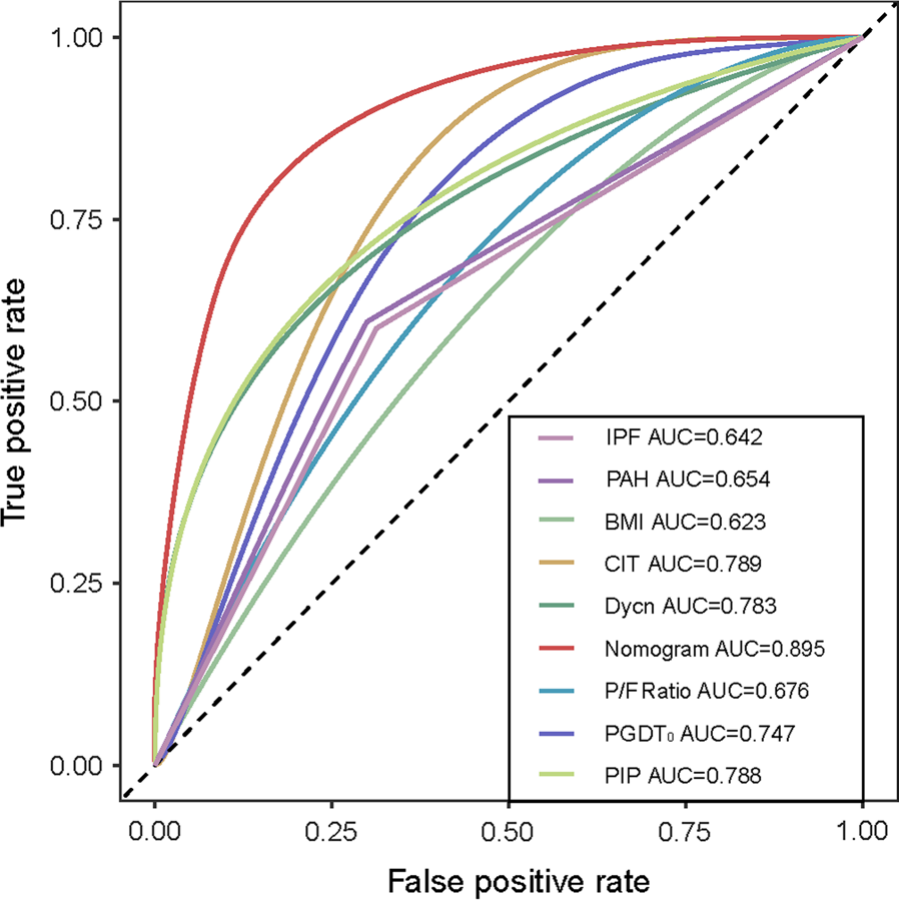
ROC analysis for the nomogram of the prediction model of recipients with prolonged mechanical ventilation after lung transplantation based on all indicators and all variables. ROC curve summation of various factors, including cold ischemia time, ventilation parameters, and PGD grade at T0. The final integrated model in the figure has an area under the ROC curve of 0.895. Among the indicators, the area under ROC curve of cold ischemia time was the largest, reaching 0.789

**Fig. 3:**
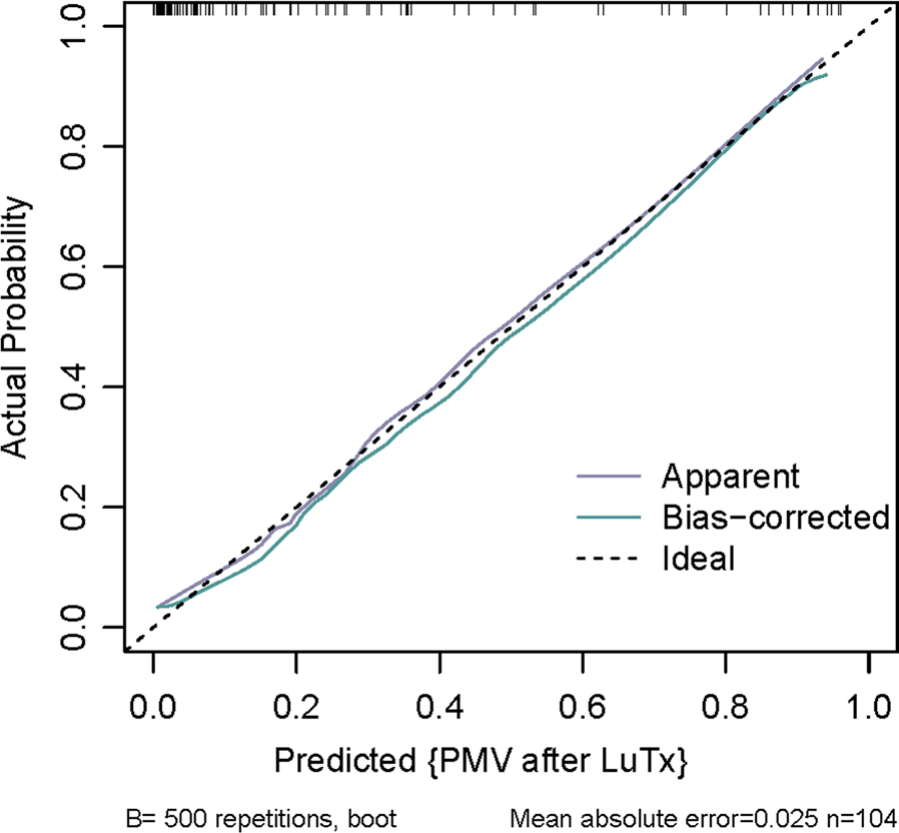
A calibration curve of this risk prediction nomogram. The model calibration has been depicted by bootstrapped calibration curve showing ideal (dotted line), apparent (purple line), and bias-corrected (green line) model

**Fig. 4:**
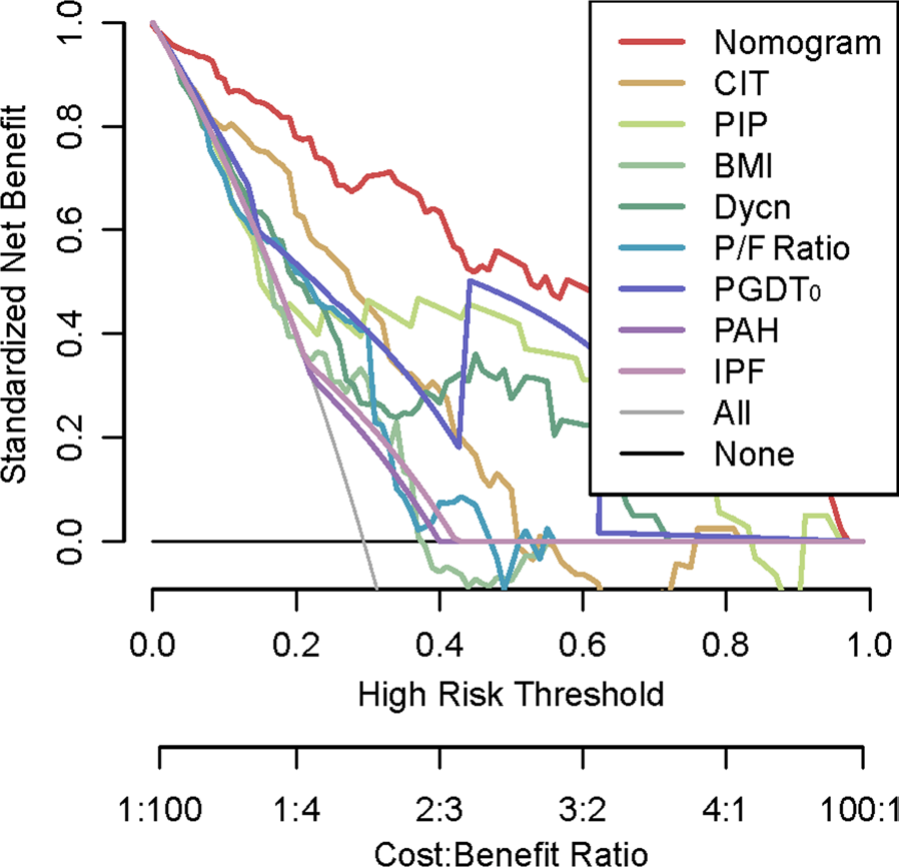
The decision curve analysis (DCA) of the prediction model of recipients with prolonged mechanical ventilation after lung transplantation based on all indicators and all variables. The prediction model or index with the largest net benefit has the best clinical guidance efficiency. Net benefit is defined as the true positive rate minus the weighted false positive rate under a given threshold probability, which defines the high risk of prolonged mechanical ventilation after lung transplantation

Based on the multivariate logistic regression, a nomogram incorporating BMI, CIT, PGD grading at T_0_, PIP, and Cdyn for predicting PMV after lung transplantation was established (Fig. 1). The model demonstrated excellent discrimination, with an AUC of 0.895 (95%CI, 0.852–0.955, Fig. 2) and an accuracy of 0.90 (Additional file 5: Table S2). A bootstrapped calibration curve was further established to estimate the predictive ability of the model, which demonstrated a superior ability with a preserved calibration. (Fig. 3). The Bootstrap test for the different ROC curves demonstrated significant differences between the nomogram and each variable included in it (*P* < 0.001). Other performance metrics are listed in the supplementary materials (Additional file 5: Table S2). As the DCA depicted in Fig. 4, the nomogram added clinical risk prediction within the range of the PMV threshold probability < 0.80, which presented satisfactory clinical usefulness. In addition, a simplified nomogram that only included four preoperative variables (pulmonary hypertension, primary diagnosis as IPF, BMI, and CIT) can be helpful in the preoperative risk assessment and early prevention of PMV (Additional file 2: Figure S2). An AUC of 0.793 also demonstrated a moderate predictive ability of this simplified nomogram (Additional file 3: Figure S3).

## Discussion

Lung transplantation is the ultimate treatment option for selected patients with end-stage lung diseases. However, the risks associated with lung transplant remain considerable. One of the most important risk factors after lung transplant is PMV, which leads to an increased cost of care and a greater risk of death for the patient [21]. Predicting patients at risk of PMV helps clinicians devise personalized care plans to mitigate the risk of PMV and timely decide on tracheostomy if ventilatory support is still required. However, tools to accurately predict PMV after lung transplant are limited. In the present study, we established a nomogram incorporating patients’ BMI, pulmonary hypertension, primary diagnosis as IPF, three ventilation parameters, CIT, and PGD grading at T_0_ to predict PMV. Compared with ventilation parameters alone, this nomogram achieved a better predictive value. Since the variables included in this nomogram are easily obtainable, the utility of this nomogram to predict the risk of PMV and guide treatment decisions may be considered routine clinical practice shortly. In more detail, lung-protective ventilation, fluid restriction, prophylactic use of ECLS, and pulmonary vasodilators may be viable options for preventing PMV in high-risk individuals from this model.

Although a series of studies have confirmed the negative prognostic impact of PMV [22, 23], the definition of PMV is still controversial, ranging from 5 h to 21 days [24]. In 2005, a report by the National Association for Medical Direction of Respiratory Care (NAMDRC) consensus conference defined PMV as mechanical ventilation for $\ge$ 21 consecutive days [25]. However, the definitional criteria may not fit all studies due to subject cohort variations. For lung transplantation, most patients undergo extubation within the first 72 h. Two previous studies defined PMV as mechanical ventilation > 72 h based on their finding that most patients (77.1% and 80.6%, respectively) were already extubated at T_72_ [3, 26]. They thus referred to > 72 h as the threshold to define PMV. A similar extubation rate (96/141, 68.1%) within the first 72 h after transplantation was observed in the present study. Therefore, we used the same criteria as in the two previously mentioned studies to define PMV.

In our study, BMI, pulmonary hypertension, primary diagnosis as IPF, PGD grading at T_0_, relevant ventilation parameters, and cold ischemia time were included in the nomogram. Obesity has long been considered an independent predictor of the length of mechanical ventilation in mechanically ventilated patients in the ICU setting [27]. Obesity and overweight are also risk factors for PGD and mortality after lung transplantation [28–30]. In the present study, although the mean BMIs in both NPMV and PMV groups do not meet the World Health Organization (WHO) criteria for overweight or obesity, however, previous reports demonstrate that Asian population develop health complications at lower BMIs than people of other races [31], and the mean BMI in the PMV group is close to the Asian-specific overweight criteria (≥ 23) [32]. Therefore, Asian patients with higher BMI should be given particular caution regarding perioperative management even though they were considered as normal weight according to international BMI chart. Thus, obese recipients may be given particular caution regarding perioperative management based on the trend for higher BMI means higher risk of PMV.

Despite previous studies that have reported that IPF and IPAH were independent predictors of increased PGD [9, 33], our findings are the first study to implicate the predictive ability of IPF as a primary diagnosis and pulmonary hypertension as a complication for early adverse events after lung transplant besides PGD. As for PGD grading at T_0_, we demonstrated that patients with NPMV were more likely to be PGD grade 0 than patients with PMV (60.4% vs. 24.4%). A previous report also revealed that patients with PGD grade 0 at T_0_ had a shorter length of mechanical ventilation than those with PGD grade 1–3 [3]. However, the AUC of PGD grading for predicting PMV was only 0.634, slightly smaller than our study (AUC = 0.747). Thus, the predictive value of PGD grading at T0 alone for PMV was limited. Although PGD grading at a later time point is reported to be more closely related to long-term outcomes after lung transplant[34], only PGD grading at T_0_ remained statistically significant in the multivariate logistic regression analysis (*P* = 0.011). A likely reason for this result is that what led to long-term outcomes did not necessarily generalize to some early outcomes, such as PMV.

The length of mechanical ventilation is closely related to the ventilation parameters [35]. Three ventilation parameters, P/F ratio, PIP, and Cdyn, were included in the nomogram in our study. Similarly, Schwarz and colleagues [3] also found these three ventilation parameters were predictors of PMV after lung transplantation. According to Ripoll et al., elevated PIP is associated with the development of acute respiratory distress syndrome (ARDS) in liver transplant recipients [36]. Moreover, Laffey et al. [37] demonstrated that higher PIP and lower P/F ratio contribute to increased hospital mortality in patients with ARDS. Cdyn was reported as a critical parameter for evaluating graft function after ex vivo lung perfusion in a previous study [38]. However, we show that in our multivariate logistic regression analysis, PIP was the strongest predictor of PMV. Only mechanical ventilation parameters at T_0_ were included in our study. This is because only ventilation parameters in the immediate postoperative period were thought to have predictive value while ventilation parameters at later times hold value for assessing the status of those patients after lung transplantation rather than being predictive.

Among these variables included in the nomogram, CIT outperformed other individual factors for predicting PMV. Since the pathological basis of PGD is consistent with IRI [39], CIT is closely related to early allograft function [40]. Recently, CIT was also reported to have a significant correlation with postoperative complications of lung transplantation [41]. However, whether CIT could be used to predict PMV remains unknown. In the present study, we demonstrated for the first time that longer CIT was an independent risk factor for PMV.

Our study has several limitations. First, one major limitation in this single-center study is that the absence of external validation may limit the application of the nomogram. Regrettably, despite repeated attempts to add a validation cohort, we ultimately failed to establish such a cohort because there are so few lung transplantation centers in China. However, both the lung transplantation centers and the annual number of lung transplants has markedly increased in recent years in China [20]. Hopefully, this preliminary result will be validated in multicenter studies in the future. Second, the sample size was relatively small. Third, the majority of the patients in our study cohort underwent a unilateral lung transplantation, which may influence the estimation of the PGD grading’s impact on length of mechanical ventilation. Although the Report of the ISHLT Working Group does not recommend separately grading PGD for bilateral and single lung transplant recipients routinely [6], previous publications do show that single lung transplantation may have an elevated overall incidence of PGD [33, 42]. In addition, the residual pulmonary function of the contralateral lung may influence the length of mechanical ventilation, which could not be evaluated in our study. Finally, this model can only be applied post-operatively to evaluate the risk for PMV after lung transplant. This may limit the interventions available to reduce the incidence of PMV and hence restricts potential applications.

## Conclusions

As shown in Visual Abstract, we established a novel nomogram that could efficiently predict individual risk of receiving PMV for patients after lung transplantation, which facilitates early diagnosis and rational intervention. Still, additional prospective validation cohorts from more clinical centers will be needed to confirm the practical utility of the newly established nomogram before its translation to wide-accepted clinical practice.

## Supplementary Information

**Additional file 1**.** Figure S1**. Study flochart.

**Additional file 2**.** Figure S2**. A simplified nomogram.

**Additional file 3**.** Figure S3**. ROC analysis for the simplified nomogram.

**Additional file 4**.** Table S1**. Detailed Ventilation Parameters of the 104 Patients at T0, T24, T48, T72.

**Additional file 5**.** Table S2**. Comparison of the performance metric for PMV.

**Additional file 6**.** Table S3**. Donor characteristics.

**Additional file 7**.** Table S4**. Primary outcomes after extubation.

**Additional file 8**.** Table S5**. Univariate logistic regression analysis testing effects of donor characteristics on predicting PMV in 141 patients after LuTx.

## Data Availability

All data that support our research will be available with the International Society for Heart and Lung Transplantation (ISHLT) following ISHLT standardized embargo and policies. The data request should be sent to the corresponding author on chenthoracic@163.com.

## Abbreviations

AUC: Area under the curve
BMI: Body mass index
Cdyn: Dynamic compliance
CI: Confidence interval
CIT: Cold ischemia time
DCA: Decision-curve analysis
ECLS: Extracorporeal life support
ECMO: Extended extracorporeal membrane oxygenation
FiO_2_: The fraction of inspiration O_2_
ICU: Intensive care unit
IRI: Ischemia–reperfusion injury
ISHLT: The International Society for Heart and Lung Transplantation
NPMV: Non-prolonged mechanical ventilation
PaO_2_: Partial pressure of oxygen
PEEP: Positive end-expiratory pressure
PGD: Primary graft dysfunction
PIP: Peak inspiratory pressure
PMV: Prolonged mechanical ventilation
ROC: Receiver operating characteristic curve
