# Supplementary figures and images for: Establishment of a risk prediction model for prolonged mechanical ventilation after lung transplantation: a retrospective cohort study
Source: BMC Pulm Med. 2023 Jan 10;23:11. doi: 10.1186/s12890-023-02307-9 (PMC9832679; doi:10.1186/s12890-023-02307-9)

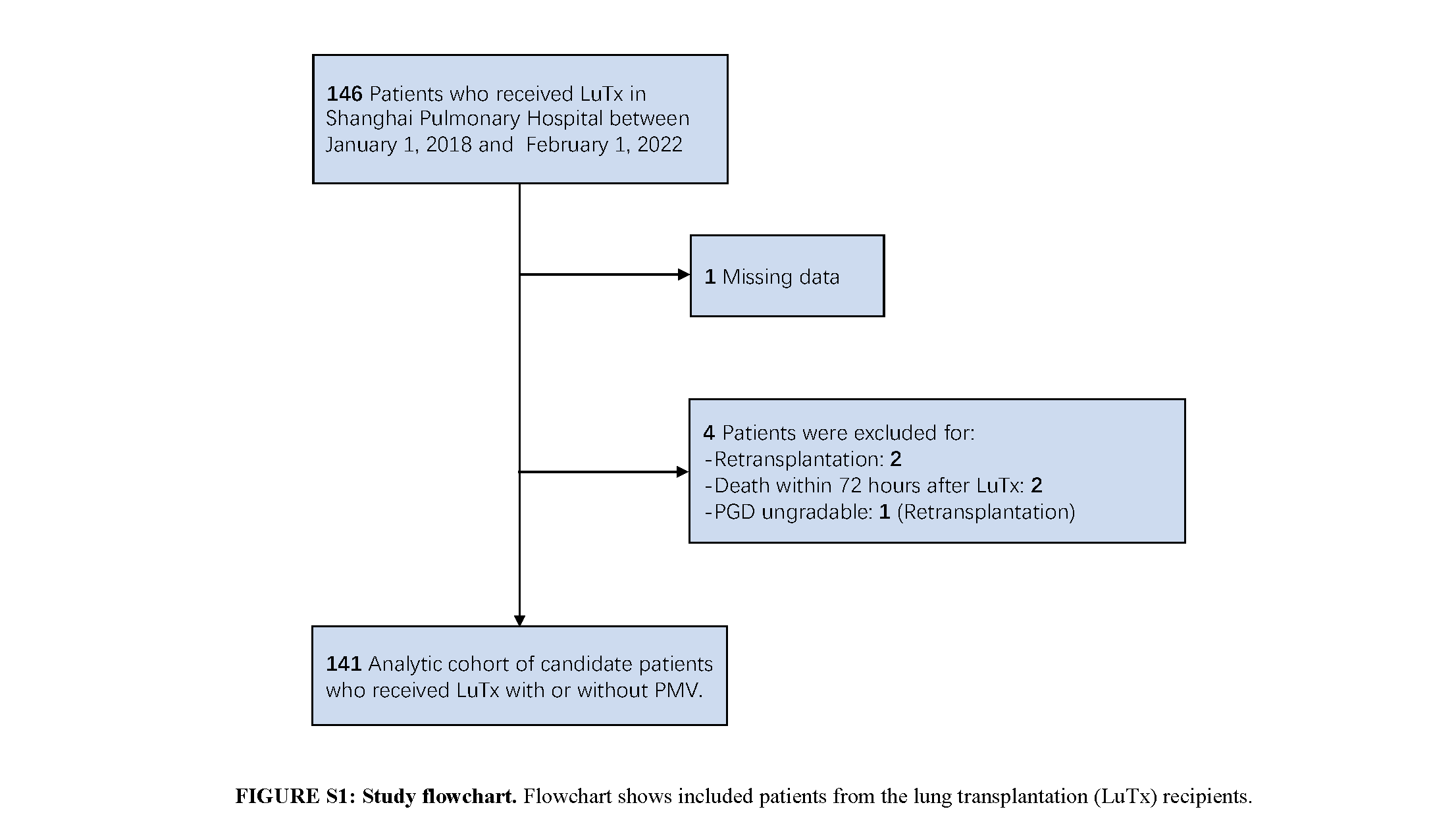

Supplement: Supplementary file 1 — Additional file 1. Figure S1. Study flochart. [file 12890_2023_2307_MOESM1_ESM.tiff]

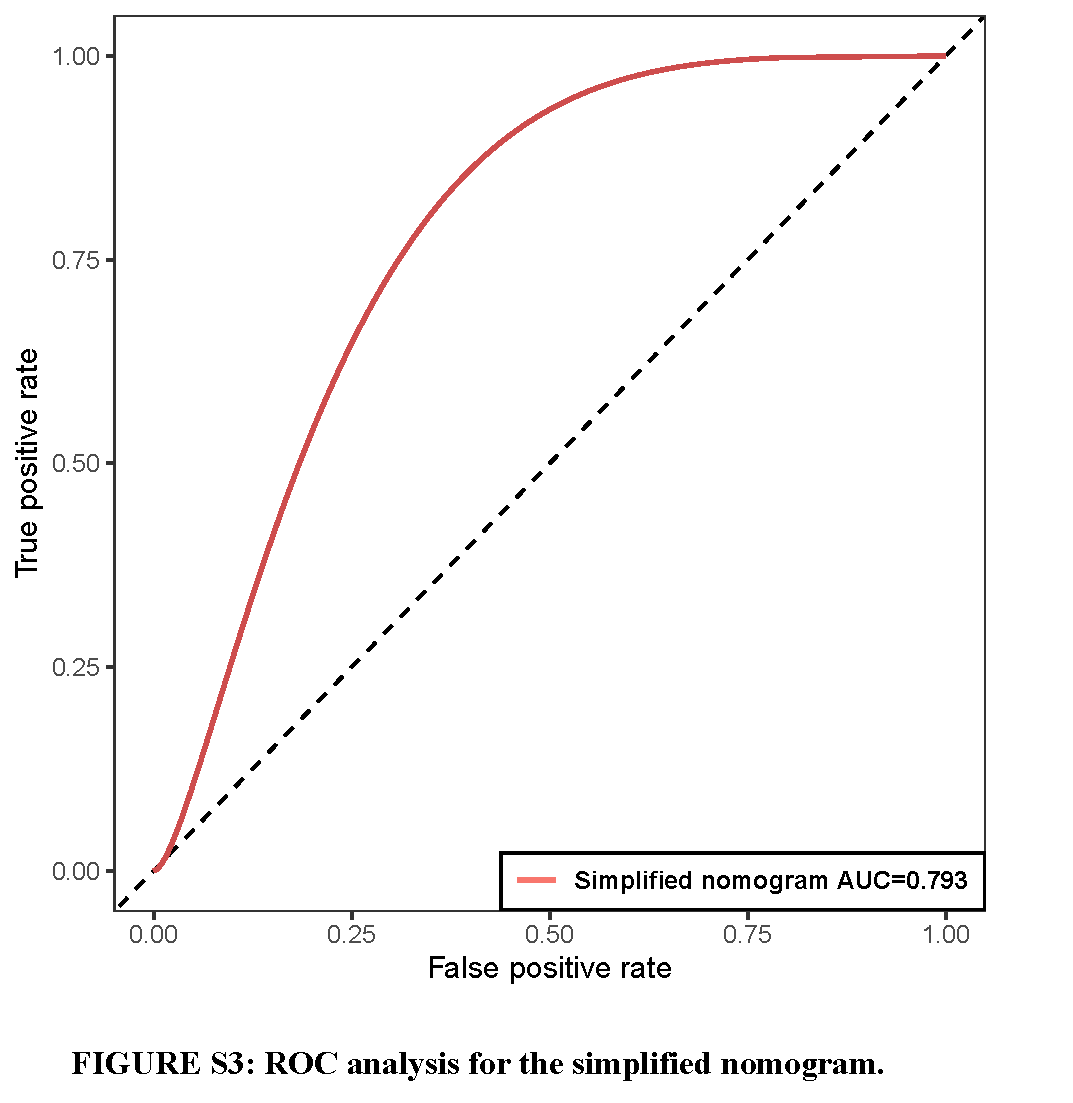

Supplement: Supplementary file 3 — Additional file 3. Figure S3. ROC analysis for the simplified nomogram. [file 12890_2023_2307_MOESM3_ESM.tiff]
