# Supplementary material for: Establishment of a risk prediction model for prolonged mechanical ventilation after lung transplantation: a retrospective cohort study
Source: BMC Pulm Med. 2023 Jan 10;23:11. doi: 10.1186/s12890-023-02307-9 (PMC9832679; doi:10.1186/s12890-023-02307-9)
Supplement: Supplementary file 4 — Additional file 4. Table S1. Detailed Ventilation Parameters of the 104 Patients at T0, T24, T48, T72. [file 12890_2023_2307_MOESM4_ESM.docx]

| Table S1. Detailed Ventilation Parameters of the 104 Patients at T0, T24, T48, T72 | | | | |
| --- | --- | --- | --- | --- |
| Parameters | T0 | T24 | T48 | T72 |
| Ventilation status, |  |  |  |  |
| Control ventilation | 114 (83.8) | 104 (76.5) | 50 (36.8) | 16 (11.8) |
| Assisted ventilation | 22 (16.2) | 32 (23.5) | 46 (33.8) | 24 (17.6) |
| Extubated | 0 (0) | 0 (0) | 40 (29.4) | 96 (70.6) |
| Unkonwn | 0 (0) | 0 (0) | 0 (0) | 0 (0) |
| Ventilation mode, |  |  |  |  |
| Pressure controlled/assisted mode |  |  |  |  |
| Pressure controlled ventilation mode | 10 (7.4) | 10 (7.4) | 20 (14.7) | 6 (4.4) |
| Pressure assisted ventilation mode | 11 (8.1) | 14 (10.3) | 21 (15.4) | 10 (7.4) |
| Volume controlled/assisted mode |  |  |  |  |
| Volume controlled ventilation mode | 104 (76.5) | 94 (69.1) | 30 (22.1) | 10 (7.4) |
| Volume assisted ventilation mode | 11 (8.1) | 18 (13.2) | 25 (18.4) | 14 (10.3) |
| Extubated | 0 (0) | 0 (0) | 40 (29.4) | 0 (0) |
| Unkonwn | 0 (0) | 0 (0) | 0 (0) | 0 (0) |
| Ventilation parameters |  |  |  |  |
| FiO_2_ | 0.60 (0.53-0.80) | 0.40 (0.40-0.45) | 0.33 (0.30-0.40) | 0.33 (0.33-0.40) |
| PEEP (cmH_2_O) | 5 (3-7) | 5 (3-8) | 5 (3-7) | 5 (3-8) |
| Peak ventilatory pressure (cmH2O) | 16 (14-20) | 15 (13-18) | 17 (12-18) | 15 (12-18) |
| Tidal volume (ml) | 390 (360-420) | 380 (370-440) | 390 (370-425) | 390 (375-420) |
| Dynamic compliance (ml/cmH2O) | 32.92 (12.56-56.76) | 28.17 (12.44-43.3) | 45.94 (21.20-56.55) | 43.33 (23.33-61.00) |
| PaO2/FiO2 ratio | 230 (150-332) | 306 (284-390) | 286 (236-366) | 291 (225-342) |
| Note: Continuous data are summarized as median and interquartile range (IQR). Categorical data are summarized as numbers and percentages. PEEP, Positive End Expiratory Pressure. | | | | |
