# Supplementary material for: Establishment of a risk prediction model for prolonged mechanical ventilation after lung transplantation: a retrospective cohort study
Source: BMC Pulm Med. 2023 Jan 10;23:11. doi: 10.1186/s12890-023-02307-9 (PMC9832679; doi:10.1186/s12890-023-02307-9)
Supplement: Supplementary file 5 — Additional file 5. Table S2. Comparison of the performance metric for PMV. [file 12890_2023_2307_MOESM5_ESM.docx]

| Table S2 Comparison of the performance metric for PMV | | | | | | | | | |
| --- | --- | --- | --- | --- | --- | --- | --- | --- | --- |
| Performance metric | Nomogram | CIT | PGDT_0_ | PIP | Cydn | BMI | P/F ratio | IPF | PAH |
| Accuracy | 0.9 | 0.715769 | 0.717308 | 0.665854 | 0.634146 | 0.588462 | 0.652128 | 0.621604 | 0.620342 |
| Sensitivity | 0.888889 | 0.962963 | 0.481481 | 0.721311 | 0.708738 | 0.402597 | 0.697433 | 0.705231 | 0.692435 |
| Specificity | 0.923077 | 0.480519 | 0.935065 | 0.553398 | 0.508197 | 0.925926 | 0.523468 | 0.514321 | 0.534576 |
| PPV | 0.857143 | 0.703939 | 0.722222 | 0.538889 | 0.708738 | 0.939394 | 0.722432 | 0.721453 | 0.716554 |
| NPV | 0.895522 | 0.973684 | 0.837209 | 0.77027 | 0.508197 | 0.352113 | 0.521810 | 0.522345 | 0.520327 |
| Note: PPV, positive predictive value; NPV, negative predictive value. | | | | | | | | | |
