# Supplementary material for: Establishment of a risk prediction model for prolonged mechanical ventilation after lung transplantation: a retrospective cohort study
Source: BMC Pulm Med. 2023 Jan 10;23:11. doi: 10.1186/s12890-023-02307-9 (PMC9832679; doi:10.1186/s12890-023-02307-9)
Supplement: Supplementary file 6 — Additional file 6. Table S3. Donor characteristics. [file 12890_2023_2307_MOESM6_ESM.docx]

| Table S3. Donor characteristics. | | | | |
| --- | --- | --- | --- | --- |
| Donor characteristics | Total  (n=141) | NPMV  (n=96) | PMV  (n=45) | *p* value |
| Age (years) | 47 (36-53) | 46 (33-52) | 49 (39-55) | 0.371 |
| Gender |  |  |  | 0.998 |
| Male | 112 (79.4) | 76 (79.1) | 36 (80.0) |  |
| Female | 29 (20.6) | 20 (20.8) | 9 (20.0) |  |
| BMI | 21.8 (20.5-22.8) | 21.3 (20.3-22.4) | 22.0 (21.1-23.1) | 0.218 |
| Smoking history |  |  |  | 0.581 |
| Never | 57 (40.4) | 37 (38.5) | 20 (44.4) |  |
| Ever | 84 (59.6) | 59 (61.5) | 25 (55.6) |  |
| Donor type |  |  |  | 0.518 |
| DCD | 40 (28.4) | 19 (19.8) | 11 (24.4) |  |
| DBD | 101 (71.6) | 77 (80.2) | 34 (75.6) |  |
| Intubation days | 4 (3-5) | 3 (2-5) | 4 (3-6) | 0.766 |
| Last ventilation parameters |  |  |  |  |
| PaO_2_ | 208 (181-262) | 229 (189-306) | 198 (160-250) | 0.089 |
| PEEP | 5 (3-7) | 4 (2-6) | 6 (4-7) | 0.455 |
| P/F ratio | 440 (382-480) | 451 (395-495) | 405 (358-460) | 0.075 |
| SaO_2_ (%) | 99.6 (99.3-100) | 99.6 (99.4-100) | 99.4 (99.2-99.9) | 0.828 |
| TV | 456 (405-520) | 460 (412-525) | 445 (398-508) | 0.612 |
| Chest X-ray |  |  |  | 0.073 |
| Infiltrates | 20 (14.2) | 10 (10.4) | 10 (22.2) |  |
| No pathological findings | 121 (85.2) | 86 (89.6) | 35 (77.8) |  |
| Bronchoscopy |  |  |  | 0.172 |
| Visible secretions | 44 (31.2) | 26 (27.1) | 18 (40.0) |  |
| No secretions | 97 (68.8) | 70 (72.9) | 27 (60.0) |  |
| Note: Continuous data are summarized as median and interquartile range (IQR). Categorical data are summarized as numbers and percentages. Abbreviations: DCD, Donation after circulatory death; DBD, Donation after brain death; SaO_2_, arterial oxygen saturation. | | | | |
