# Supplementary material for: Establishment of a risk prediction model for prolonged mechanical ventilation after lung transplantation: a retrospective cohort study
Source: BMC Pulm Med. 2023 Jan 10;23:11. doi: 10.1186/s12890-023-02307-9 (PMC9832679; doi:10.1186/s12890-023-02307-9)
Supplement: Supplementary file 7 — Additional file 7. Table S4. Primary outcomes after extubation. [file 12890_2023_2307_MOESM7_ESM.docx]

| Table S4. Primary outcomes after extubation. | | | | |
| --- | --- | --- | --- | --- |
| Variables | Total  (n=141) | NPMV  (n=96) | PMV  (n=45) | *p* value |
| Ventilation strategies |  |  |  |  |
| NIV | 49 (34.8) | 29 (30.2) | 20 (44.4) | 0.272 |
| HFNC | 52 (36.9) | 34 (35.4) | 18 (40.0) | 0.325 |
| Venturi mask | 28 (19.9) | 22 (22.9) | 6 (13.3) | 0.164 |
| Others | 12 (8.5) | 9 (9.4) | 3 (6.7) | 0.628 |
| Reintubation | 32 (22.7) | 15 (15.6) | 17 (37.8) | **0.046** |
| Note: Continuous data are summarized as median and interquartile range (IQR). Categorical data are summarized as numbers and percentages. Abbreviations: NIV, Noninvasive ventilation; HFNC, High-flow nasal cannula oxygen therapy. | | | | |
