# Supplementary material for: Establishment of a risk prediction model for prolonged mechanical ventilation after lung transplantation: a retrospective cohort study
Source: BMC Pulm Med. 2023 Jan 10;23:11. doi: 10.1186/s12890-023-02307-9 (PMC9832679; doi:10.1186/s12890-023-02307-9)
Supplement: Supplementary file 8 — Additional file 8. Table S5. Univariate logistic regression analysis testing effects of donor characteristics on predicting PMV in 141 patients after LuTx. [file 12890_2023_2307_MOESM8_ESM.docx]

| Table S5. Univariate logistic regression analysis testing effects of donor characteristics on predicting PMV in 141 patients after LuTx | | | |
| --- | --- | --- | --- |
| Donor Characteristic | Univariable | | |
|  | OR | 95% CI | p value |
| Age, y | 1.356 | 1.281 -1.508 | 0.468 |
| BMI | 1.102 | 1.011-1.265 | 0.309 |
| Gender | 1.563 | 1.337-1.775 | 0.167 |
| Smoking history  Nonsmoker vs Smoker | 1.147 | 1.045-1.301 | 0.542 |
| Donor Type  DBD vs DCD | 1.821 | 1.437-2.224 | 0.210 |
| Intubation days | 2.126 | 1.177-2.405 | 0.121 |
| Last PaO_2_ | 1.367 | 1.201-1.525 | 0.334 |
| Last PEEP | 1.213 | 1.041-1.436 | 0.205 |
| Last P/F ratio | 1.187 | 1.027-1.327 | 0.125 |
| Last SaO_2_ | 1.542 | 1.346-1.874 | 0.194 |
| Last TV | 1.765 | 1.408-2.090 | 0.369 |
| Chest X-ray  Infiltrates vs No pathological findings | 2.563 | 1.352-3.568 | 0.103 |
| Bronchoscopy  Visible secretions vs No secretions | 2.875 | 1.998-3.642 | 0.110 |
| Note: Continuous data are summarized as median and interquartile range (IQR). Categorical data are summarized as numbers and percentages. Abbreviations: DCD, Donation after circulatory death; DBD, Donation after brain death; SaO_2_, arterial oxygen saturation. | | | |
